# Spontaneous Tension Hemothorax in a Patient With Neurofibromatosis Type 1

**DOI:** 10.7759/cureus.71486

**Published:** 2024-10-14

**Authors:** Yuki Koike, Daiyu Kousen, Takayuki Kurinobu, Keiki Shimizu

**Affiliations:** 1 Department of Emergency and Critical Care Medicine, ECMO Center, Tokyo Metropolitan Tama Medical Center, Tokyo, JPN; 2 Department of Radiology, Tokyo Metropolitan Tama Medical Center, Tokyo, JPN

**Keywords:** hemorrhage, shock, thoracic cavity, transcatheter arterial embolization, von recklinghausen’s disease

## Abstract

Neurofibromatosis type 1 (NF1), also known as von Recklinghausen disease, is the most common phacomatosis. It is characterized by neurofibromas but also manifests vascular complications including stenosis, arterial ectasia, and aneurysms. We report the case of an otherwise healthy 45-year-old male who developed a fatal tension hemothorax due to bleeding from the left costocervical artery. Tension hemothorax without trauma is rare except in cases with a rupture of an aortic aneurysm; we successfully treated the patient by promptly diagnosing tension hemothorax and performing chest drainage, as well as timely transcatheter arterial embolization (TAE) to control bleeding. When encountering shock patients with NF1, clinicians should be mindful of the potential for non-traumatic hemorrhagic complications due to vascular malformation.

## Introduction

Neurofibromatosis type 1 (NF1), the most common phacomatosis, was first described by von Recklinghausen in 1882 [1]. NF1 is characterized by neurofibromas but also manifests other organ lesions such as musculoskeletal abnormalities, skin disorders, and vascular disorders [2]. Vascular lesions are less well-documented in NF1, with reports indicating that they occur in only 1-3% of cases [3]. They can present in various ways, including stenosis, aneurysm formation, arteriovenous fistula, vascular proliferation, and invasion, or compression by neural tumors. One of the symptoms of NF1 vascular disorders is spontaneous haemothorax, which is one of the more severe manifestations of NF1, with a low incidence and the potential for fatal outcomes [4]. While spontaneous hemothorax does not involve a traumatic mechanism, it can lead to tension hemothorax in some cases, and there have been reports of fatalities associated with this condition [5]. We discuss a case of a patient with NF1 admitted for spontaneous tension hemothorax.

## Case presentation

A 45-year-old male with a history of NF1 presented to the emergency department with sudden-onset dyspnea. He had been in his usual state of health until three days before the presentation when he noticed discomfort in his left shoulder. He had a history of NF1 and had been followed up as an outpatient at another hospital. He had no history of trauma and was neither on anticoagulant nor antiplatelet therapy. At his admission to the emergency department, he was hemodynamically unstable and in respiratory distress. His vital signs were as follows: respiratory rate of 24 breaths per minute, a heart rate of 130 beats per minute, a blood pressure of 54/28 mmHg, a body temperature of 35 °C, and oxygen saturation level of 95% on O_2_ 10L. Physical examination revealed absent breathing sound of the left lung and jugular vein distension.

Laboratory evaluation revealed white blood cell counts (WBCs) of 13,600/mm^3^, hemoglobin of 12.2 g/dl, red blood cell counts (RBCs) of 4.08 × 10^6^/mm^3^, platelet counts of 185 × 10^3^/mm^3^, creatinine of 1.4 mg/dl, prothrombin time of 12.9 seconds (reference range: 9.8-12.1 seconds), and activated partial thromboplastin time of 21.6 seconds (reference range: 23.5-31.5 seconds) (Table 1).

**Table 1:** Laboratory reports Significant laboratory findings were as follows: white blood cell counts (WBCs) of 13600/mm^3^, hemoglobin of 12.2 g/dl, red blood cell counts (RBCs) of 4.08 × 10^6^/mm^3^, platelet counts of 185 × 10^3^/mm^3^, creatinine of 1.4 mg/dl, prothrombin time of 12.9 seconds, and activated partial thromboplastin time of 21.6 seconds

Lab parameters	Observed value	Reference range
Hemoglobin, g/dL	12.2	12.1-15.1
Total leucocyte count, x 10^3^/µL	13.6	4.0-11.0
Platelets, x 10^3^/µL	185	150-450
Red blood cell, x 10^6^/µL	4.08	4.0-5.0
Prothrombin time, seconds	12.9	9.8-12.1
International normalized ratio	1.17	0.8-1.2
Activated partial thromboplastin time, seconds	21.6	23.5-31.5
Fibrinogen, mg/dL	250	155-415
Urea, mg/dL	14.7	8.0-20.0
Creatinine, mg/dL	0.71	0.65-1.07
Alkaline phosphatase, IU/L	49	38-113
Alanine transaminase, U/L	23	10-42
Aspartate aminotransferase, U/L	24	13-30
Total bilirubin, mg/dL	1.1	0.4-1.5
Albumin, g/dL	3.4	4.1-5.1
pH	7.404	7.350-7.450
pO_2_, mmHg	51.4	80.0-100.0
pCO_2_, mmHg	42.1	35.0-45.0
HCO_3_, mmol/L	25.8	22-26
Na, mmol/L	138	136-145
K, mmol/L	3.8	3.5-4.5
Cl, mmol/L	106	98-107
Ca, mmol/L	1.16	1.15-1.33
Glucose, mg/dL	106	65-95
Lactate, mmol/L	1.5	0.4-0.8

**Figure 1:**
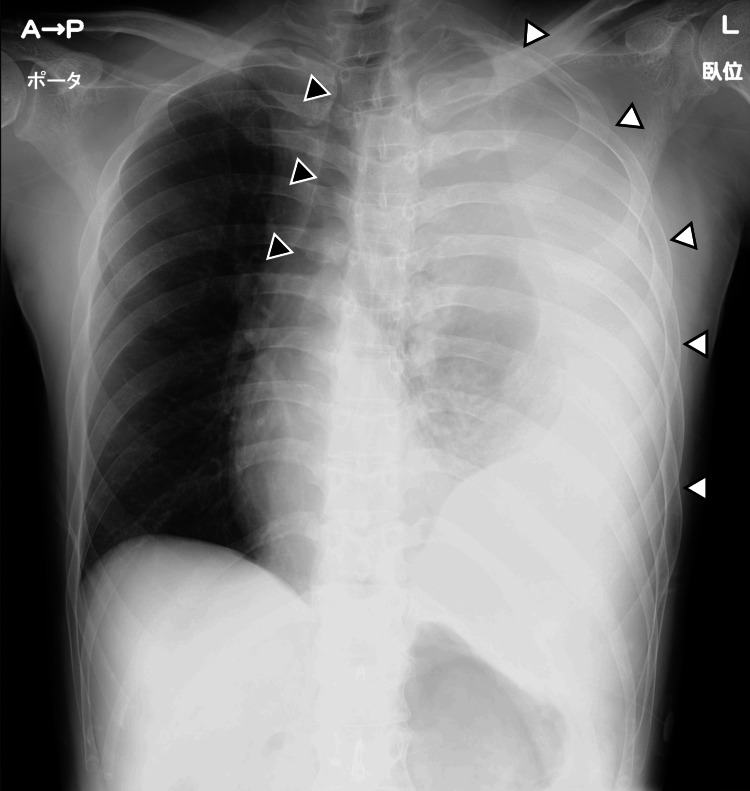
Chest X-ray Complete opacification of the left thorax (white arrows) and mediastinal shift toward the right (black arrows)

Chest X-rays showed total opacification of the left thorax, accompanied by a mediastinal shift toward the right (Figure 1).

**Figure 2:**
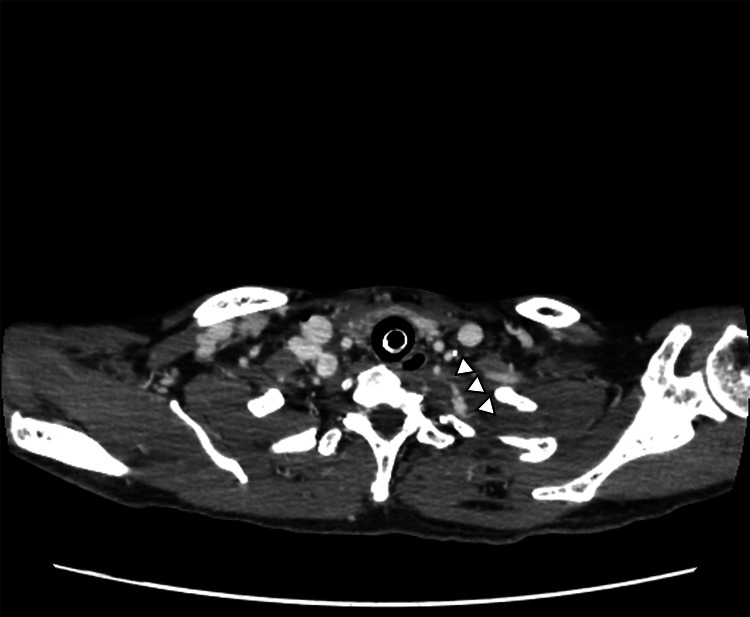
Contrast-enhanced CT Hemothorax on the left chest cavity with extravasation (white arrows) in hematoma on the pulmonary apex region CT: computed tomography

As the patient’s blood pressure was unresponsive to fluid administration and noradrenalin, intubation and thoracic cavity drainage were performed. After draining 1,500 milliliters of hemorrhagic pleural effusion by a chest tube insertion into the left thorax, his blood pressure recovered to 110/50 mmHg. After hemodynamics were stabilized, contrast-enhanced CT (CECT) was performed, which revealed massive hemothorax on the left chest cavity with extravasation in hematoma on the pulmonary apex region (Figure 2).

**Figure 3:**
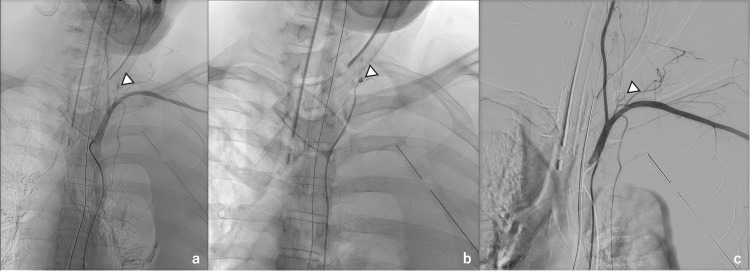
Angiography Angiography of the left subclavian artery revealed a pseudoaneurysm in the left costocervical artery (a,b). The pseudoaneurysm was embolized using n-butyl-2-cyanoacrylate (c)

Transcatheter arterial embolization (TAE) using the micro-coaxial catheter technique was performed after a transfusion of six units of RBC and six units of fresh-frozen plasma (FFP). Angiography of the left subclavian artery revealed a pseudoaneurysm in the left costocervical artery, which was embolized using n-butyl-2-cyanoacrylate (Figure 3).

TAE improved the patient's hypotension and anemia, and he was extubated three days after admission. The chest tube was removed six days after admission. The patient was fully recovered and discharged after eight days of hospitalization.

## Discussion

NF1, first described by von Recklinghausen in 1882, is the most common phacomatosis [1]. The incidence of NF1 is estimated to be 1:2600 to 1:3000 individuals. Around 50% of the cases are inherited [6]. The genes responsible for NF1 are pathogenic variants in the NF1 gene, which is located on chromosome 17q11.2 [7]. Clinically, NF1 is typically characterized by café-au-lait macules, axillary and/or inguinal freckling, Lisch nodules, and neurofibromas. Vascular lesions are less common in NF1 (1-3% of cases) [3]. Vascular lesions reported include arterial stenosis, aneurysms, spontaneous arterial rupture, and arteriovenous fistulae. Several mechanisms have been proposed regarding the pathogenesis of vascular lesions in patients with NF1. It is believed that large vessels are directly infiltrated by neighboring tumors, such as schwannomas, leading to intimal proliferation, media thinning, and fragmentation of elastic tissue. These changes can result in stenosis or the formation of aneurysms. It is believed that smaller vessels exhibit wall dysplasia characterized by fibro-hyaline thickening of the intima and muscularis, leading to stenosis and a consequent marked weakening of the arterial wall [8].

Reports of massive hemothorax in NF1 patients are rare. Spontaneous hemothorax, a rare but often fatal complication of NF1, may arise from the bleeding of dysplastic small vessels or the rupture of an aneurysm in a major intrathoracic artery. The mortality rate for such events is reported to be up to 36%, and the operative mortality rate is reported to be 33% [4]. It is noteworthy that this case involved not only massive hemothorax but also tension hemothorax. A tension hemothorax is a serious condition that can cause obstructive shock and, if not treated promptly, can result in cardiac arrest.

This report involves the second case of a tension-type hemothorax due to NF1, following the report by Bidad et al. [5]. We believe that our patient's survival was aided significantly by the prompt diagnosis of tension hemothorax and chest drainage, which prevented cardiac arrest, and timely TAE to control bleeding. In the case of an NF1 patient who is in shock, it is important to consider the possibility of non-traumatic hemorrhagic complications due to vascular malformations, as this enables a rapid response to a potentially fatal condition.

## Conclusions

Spontaneous hemothorax is an extremely rare but potentially life-threatening complication in patients with NF1 and should be taken seriously during the management of NF1 patients. The prompt diagnosis of tension hemothorax and chest drainage and timely TAE to control bleeding can save these patients. All ER physicians should be aware that in patients with NF1 who are in shock, there is a potential for non-traumatic hemorrhagic complications due to vascular malformation.
